# Centenarianism

**Published:** 1905-07-29

**Authors:** 


### Centenarianism.

The accepted physiological doctrine, that the life of man in these latitudes would normally extend to one hundred years, and that its ordinary curtailment is due to causes which, at least in hypothesis, are preventible, has furnished Sir James Crichton Browne with the text of a highly interesting discourse. His audience at one of the meetings of the London Conference on Public Health received from him an assurance, not only that the male sex could accomplish its " century " in a sense differing from that of the cricket field, but that its female helpmates could do still more, and that they are naturally calculated to live longer than men. The assumption is, we are inclined to think, to some extent attributable to " the gallantry of man, in lovelier woman's cause," for it is at least probable that the ordinary greater longevity of women than of men is chiefly due to the more sheltered and more temperate lives of the former, and is, therefore, accidental rather than physiological. If both sexes ordinarily reached the full term of natural existence the difference would probably cease to be apparent. Putting this question aside, as one on which our experience is too limited to permit of a conclusion, there can be no doubt that Sir James is fully justified in his assertion that people generally ought to live longer than they do, and that they are to some extent responsible for the premature curtailment of existence. If we turn to existing records of longevity we shall find that the most conspicuous examples have been derived from classes of persons who, at first sight, appear to have only few common characteristics. Great poets and great philosophers (especially great mathematicians) have frequently not only attained great age, but have preserved their intellectual faculties in high activity to the last; while longevity in an almost equal degree has been displayed by people, especially by women in the humblest ranks of life. There is scarcely a country village which does not contain its old Betty or its old Sally, living in one room on a pittance of extreme tenuity, and full of anecdotes about the youth of the grandparents of the existing generation. These old women have led laborious lives, often as farm servants, and as the wives and mothers of men of the labouring class, and they have been preserved from every kind of excess, not only by poverty, but by the honest pride of respectability and of good conduct. The drugged beer of the village alehouse has not numbered them among its victims; and the maxim of the last generation, to rise from table with an appetite, is one which they have observed from necessity as much as from good manners. The lesson which their long lives are calculated to teach is much the same as that which was taught by Cornaro—namely, that human requirements in the way of food are much smaller than is commonly supposed, and that the labour of disposing of excess is much more than a compensation for any support which may be derived from consuming it. On the other hand, the longevity of the philosopher tends to drive home a doctrine which Sir James Crichton Browne has often taught, the doctrine, that is, of a close correspondence between the healthy development of the brain and that of the organs which are subordinate to it. He has often pointed out, with regard to education, that, if certain groups of muscles be left unexercised, the motor centres connected with them will not attain their normal development, and that the brain generally is likely to suffer from the consequent sub-nutrition of some of its constituent parts. The principle is of wide application, and it involves the consequence that an exercised and highly-nourished brain implies a corresponding condition of all the organs to which nerves are supplied, and which perform functions that are largely under nervous control. Moreover, the business and the pleasure of the philosopher are to think, and to think clearly; and for these purposes it is essential that he should neither overload his digestive system with food nor muddle his brain with alcohol.

